# Regression Model Decreasing the Risk of Femoral Neurovascular Bundle Accidental Puncture

**DOI:** 10.3390/tomography8050208

**Published:** 2022-10-01

**Authors:** Juan Antonio Valera-Calero, Umut Varol, Gustavo Plaza-Manzano, César Fernández-de-las-Peñas, Adolfo Agudo-Aguado

**Affiliations:** 1 Department of Physiotherapy, Faculty of Health, Universidad Camilo José Cela, 28692 Madrid, Spain; 2 VALTRADOFI Research Group, Department of Physiotherapy, Faculty of Health, Universidad Camilo José Cela, 28692 Madrid, Spain; 3 Department of Radiology, Rehabilitation and Physiotherapy, Universidad Complutense de Madrid, 28040 Madrid, Spain; 4 Instituto de Investigación Sanitaria San Carlos (IdISSC), 28040 Madrid, Spain; 5 Department of Physical Therapy, Occupational Therapy, Rehabilitation and Physical Medicine, Universidad Rey Juan Carlos, 28922 Alcorcón, Spain; 6 Cátedra Institucional en Docencia, Clínica e Investigación en Fisioterapia: Terapia Manual, Punción Seca y Ejercicio Terapéutico, Universidad Rey Juan Carlos, 28922 Alcorcón, Spain

**Keywords:** sartorius muscle, dry needling, ultrasound imaging, accident prevention, clinical decision rules

## Abstract

Although most of the adverse events derived from dry needling are minor, avoiding potential hazards for patients including accidental invasion of vessels, ganglia, and nerves is essential to ensure patients’ safety. We aimed to investigate the contribution of predictors explaining the variance of sartorius muscle depth limit at proximal third and middle thigh as these locations lead to an augmented risk of neurovascular bundle invasion during dry needling application. A diagnostic study was conducted on 84 subjects to calculate the accuracy of a prediction model for sartorius depth, as assessed with ultrasound imaging, based on sex, age, height, weight, body mass index (BMI), thigh perimeter, and length. After calculating a correlation matrix, a multiple linear regression analysis was performed to detect those variables contributing to the sartorius deep limit in both locations. Although males showed greater thigh perimeter than women (*p* < 0.001), the deep limit of the sartorius muscle was significantly more superficial for both the proximal third (*p* = 0.003) and the mid-third (*p* = 0.004) points. No side-to-side anthropometric differences were found (*p* > 0.05). In addition, we found sartorius muscle depth to be associated with the proximal and mid-third girth, gender, height, and BMI (all, *p* < 0.01). Gender, proximal-third girth, and BMI explained 51.1% and 42.6% of the variance for the sartorius deep limit at the proximal and the mid-third, respectively. This study analyzed whether anthropometric features could predict sartorius muscle depth in healthy participants for assisting clinicians in choosing the optimal needle length to avoid accidental femoral bundle puncture.

## 1. Introduction

The sartorius is the longest muscle in the body and the most superficial muscle in the anterior compartment of the thigh. It originates from the anterior superior iliac spine and inserts into the proximomedial tibia, below the tibial tuberosity, associated with the gracilis and semitendinosus tendons (pes anserinus) [1,2]. It lies immediately lateral to the femoral neurovascular bundle (femoral nerve, artery, and vein), forming the lateral border of the femoral triangle [2]. Further, the sartorius muscle covers the adductor canal (subsartorial canal), a musculoaponeurotic tunnel on the medial aspect of the distal two-thirds of the thigh. The femoral artery and vein, saphenous nerve, posterior branch of the obturator nerve, and, in some cases, medial retinacular nerve or medial femoral cutaneous nerve are contained in the adductor canal [3,4,5]. The sartorius muscle is innervated by the femoral nerve and contributes to hip external rotation and flexion, knee internal rotation and flexion, as well as being a secondary valgus stabilizer of the knee [1,2].

A musculoskeletal impairment in the sartorius muscle could lead to biomechanical changes in the hip and knee. This muscle can be affected in pes anserinus tendinopathy or bursitis and posteromedial knee friction syndrome. In fact, its location predisposes it to strain and blunt concussive injuries [1]. However, myofascial trigger points (MTrPs) are considered one of the most common muscle impairments observed in the lower extremity. An MTrP is defined as a hypersensitive spot within a taut band of a skeletal muscle that is painful with mechanical stimulation (e.g., palpation, contraction, or needling), elicits a referred pain, and induces motor disturbances [6]. Active MTrPs elicit referred pain that reproduces the symptoms of a patient, whereas MTrPs are latent when referred pain does not reproduce any symptom [6]. In fact, the prevalence of active MTrPs in the sartorius muscle is 57.6% in individuals with patellofemoral pain syndrome [7] and 64.8% in those with painful knee osteoarthritis [8].

Dry needling and manual therapy are the most common therapeutic interventions used in the management of MTrPs. Dry needling refers to the insertion of a solid filiform needle into the MTrP and has received increasing attention in the recent literature [9,10,11]. Dry needling of the sartorius muscle, among other knee musculature, showed significant improvements in pain and disability in individuals with knee osteoarthritis [12]. Since dry needling is an invasive technique, improving its safety is essential [13,14].

Although dry needling is considered a safe intervention, adverse events have been described in the literature. Most adverse events related to dry needling can be considered minor (e.g., bruising, bleeding, and pain during or after treatment); however, serious adverse events (e.g., lasting nerve damage) can also occur [13,15]. A previous study [16] reported an estimated risk rate of ≤0.04% for significant adverse events.

Due to the anatomical location of the sartorius muscle and its relationships to the femoral neurovascular bundle and structures included in the adductor canal, proper safety and prevention are needed. In fact, various strategies have been proposed to ensure patient safety and reduce relative risk during invasive procedures, such as patient positioning [14] or ultrasound imaging [17]. Previous studies [17,18] have recommended ultrasound-guided dry needling to ensure the correct needle location and depth of penetration in some muscles, e.g., rhomboid or quadratus lumborum, located close to important anatomical structures. However, the routine use of ultrasound imaging in regular clinical practice is not always possible due to high economic costs.

A recent study has developed a prediction model based on calf anthropometric measures for assessing the necessary needle length to prevent tibial nerve injury during the application of dry needling in the soleus muscle [19]. A similar procedure could help in reducing the risk of puncturing the femoral neurovascular bundle when needling the sartorius muscle. Therefore, this study aimed to evaluate if anthropometric features can predict sartorius muscle depth, assessed with ultrasound imaging, in a sample of healthy subjects. Our hypothesis is that a prediction model based on anthropometric features of the thigh could assist during the application of dry needling into the sartorius muscle.

## 2. Materials and Methods

### 2.1. Study Design

This is a cross-sectional observational study (diagnostic accuracy) to calculate a regression model for predicting the distance between the skin and the sartorius muscle deep limit (assessed with US) in two locations with a high risk of accidental vasculonervous bundle invasion based on anthropometric features including age, height, weight, body mass index (BMI), gender, and thigh length and perimeter. This study followed the Standards for the Reporting of Diagnostic Accuracy Studies (STARD) guidelines and checklist [20].

### 2.2. Participants

A consecutive sample consisting of volunteers (recruited by using local announcements in a private university located in Madrid, Spain) participated in this study. To be eligible to participate, they had to be between 18 and 65 years old.

Participants under pharmacological treatment affecting muscle tone (e.g., muscle relaxants or analgesics); having a prior history of lower limb surgery, and any other medical condition such as a tumor, fracture, or other neuromuscular condition compromising normal lower limb morphology (e.g., sarcopenia, myasthenia gravis, amyotrophic lateral sclerosis, or multiple sclerosis) were excluded.

This study was approved by the Institutional Ethics Committee of Camilo José Cela University. All participants read and signed a written informed consent prior to their participation in this study.

### 2.3. Sample Size Calculation

Based on a previous study with a similar design (regression models based on anthropometric features applied to the lower extremity to prevent adverse effects derived from dry needling) [19], a sample size of at least 96 measurements was considered. In addition, considering (1) the recommendations of including a range from 10 to 15 subjects per potential predictor [21] to obtain a proper sample size for prediction models and for avoiding overestimation of the results; and (2) that our study design appraises no more than eight predictor variables in the model (age, gender, height, weight, BMI, thigh circumference, length, and side), we estimated a minimum sample size of 80 participants.

### 2.4. Procedure

Anthropometric data included age, gender, height, weight, and BMI. Further, thigh perimeter, sartorius muscle length, and side were evaluated. Firstly, length was measured as the distance between its origin (ASIS) and its insertion (pes anserine) with US guiding.

**Figure 1:**
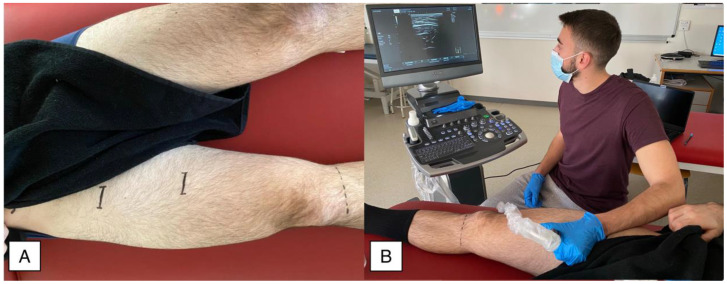
(**A**) Location of the examination point and (**B**) Probe placement during the assessment.

Patients were placed supine on a table with their knees extended, ankles positioned and stabilized with a rigid table at 0° of dorsiflexion and lower extremity muscles resting. Measurement points to calculate the thigh perimeter consisted of the proximal third and middle distance between those reference points (Figure 1) as the sartorius muscle is in close contact with the femoral vessels and MTrPs are common in these areas [22].

**Figure 2:**
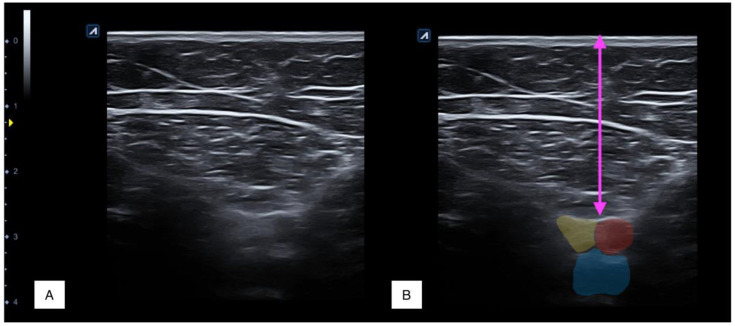
Ultrasound imaging of sartorius muscle at the proximal third (**A**). The pink arrow indicates the distance between the skin and the deep sartorius fascia limit. The femoral bundle is colored in yellow (nerve), red (artery), and blue (vein) (**B**).

For ultrasound imaging, participants were in the same position. A single examiner with +10 years of experience in US imaging conducted all imaging procedures bilaterally using an Alpinion Ecube 8 US equipment with a linear transducer E8-PB-L3-12T 3-12 MHz (Gyeonggi-do, Korea). Ultrasound parameters were individually modified for each imaging capture to optimize the imaging quality. To obtain the image, the examiner placed the transducer considering the minimal pressure possible until locating the sartorius muscle in both measurement points and finally measuring the distance between the skin and the internal interface of the deep sartorius limit (Figure 2). To improve the accuracy of measurements, a mean of three trials was calculated for the main analysis.

### 2.5. Statistical Analysis

All analyses were conducted using the Statistical Package for the Social Science (SPSS) V.25 (Armonk, NY, USA) for Mac OS. Normal distribution was verified by running a Saphiro–Wilk test (normal if *p* > 0.05). After verifying the sample homogeneity, Student’s t-tests for independent samples were used for analyzing gender (i.e., age, height, weight, BMI, muscle length, and skin-to-sartorius deep limit distance and thigh perimeter at both measurement points) and side (muscle length, thigh perimeter, and US variables) differences.

A Pearson’s correlation matrix was calculated to determine the association between normal distributed variables. Correlation coefficients (r) were interpreted as poor (*r* < 0.3), fair (0.3 < *r* < 0.5), moderate (0.6 < *r* < 0.8), or strong (*r* > 0.8) [23]. In addition, *r* was used to identify multicollinearity and shared variance (if *r* > 0.80) to avoid the risk of bias and overestimation of the calculated model.

Finally, those variables showing statistically significant correlations (*p* < 0.05) with sartorius depth were included in a forward stepwise multiple linear regression model to estimate the ultrasound distance. The significance criterion of the critical F value for entry into the regression equation was set as *p* < 0.05. The adjusted changes in R2 were reported step by step in the regression model to determine the association of each additional variable.

## 3. Results

Of 90 volunteers responding to the announcements, six were excluded due to severe lower limb asymmetries. Finally, 84 subjects (58.3% males) were included. After assessing both sides for each subject, a total of 336 measurements (84 measurements per side in the proximal and the middle thigh third) were analyzed.

Sociodemographic data of the sample by gender and side are summarized in Table 1. Men were significantly older (*p* < 0.05), taller (*p* < 0.01), heavier (*p* < 0.01), and more overweight (*p* < 0.05) than women.

Anthropometric analyses are summarized in Table 2. Although men had larger thighs (*p* < 0.001), girth was comparable between men and women (*p* > 0.05). In addition, the sartorius muscle deep limit was significantly more superficial in men at the proximal third (*p* = 0.003) and the mid-third (*p* = 0.004). No side-to-side differences for the anthropometric features were found.

Table 3 reports Pearson’s correlation coefficients of sartorius muscle depth with sociodemographic and anthropometric features. Sartorius depth was shown to be positively associated with thigh girth at the proximal and mid-third (*p* < 0.01), female sex (*p* < 0.01), BMI (*p* < 0.01), and height (*p* < 0.01).

Table 4 describes the hierarchical regression analysis conducted to determine sartorius muscle depth at the proximal third of the thigh. Gender, proximal-third thigh girth, and BMI contributed up to 51.1% of the variance for the proximal-third location and up to 42.6% of the variance for the mid-third. An alternative model including height instead of BMI is detailed in Supplementary Materials (Table S1).

**Figure 3:**
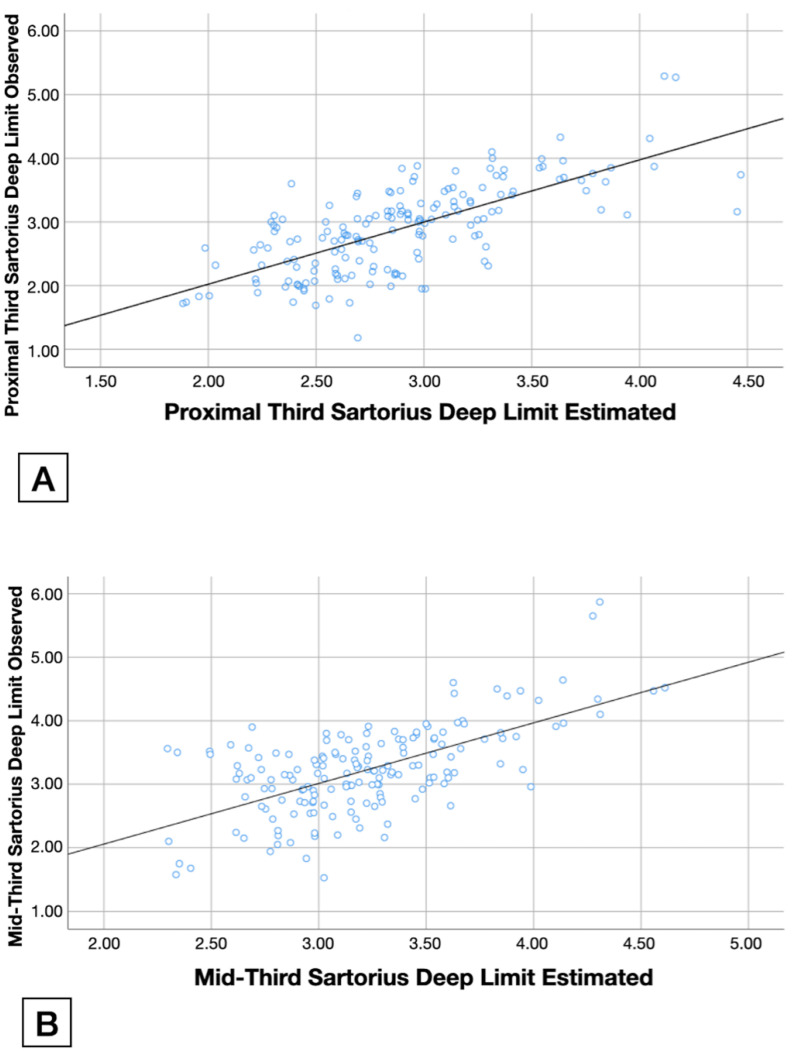
Real (*Y*-axis) and estimated (*X*-axis) values of sartorius deep limit in cm at the proximal third (**A**) and mid-third (**B**). Blue points represent each participant and black lines represent the tendency.

As a result of this multiple linear regression model, Figure 3 shows the observed values for the sartorius muscle deep limit (Figure 3A at the proximal third and Figure 3B at the mid-third) in the *Y*-axis, the estimated value calculated with this model in the *X*-axis, and the tendency lines.

## 4. Discussion

This study found that sociodemographic and anthropometric features were able to predict sartorius muscle deep limit in healthy individuals. Current findings could assist clinicians during needling procedures to reduce the risk of accidental femoral bundle puncturing by determining the most appropriate needle length without an ultrasound guide.

Although this is not the first study conducting regression models to decrease the risk of adverse events during the application of needling techniques applied to musculoskeletal structures predicting the optimal needle length [19,24], this is the first report calculating a specific model for the sartorius muscle.

The rationale for conducting this study was based on previous research supporting the impact of MTrP on the sartorius muscle in clinical conditions affecting the knee [25]. For instance, a previous report analyzing whether referred pain elicited by active MTrPs reproduced symptoms in patients with knee osteoarthritis found a positive correlation between the presence of MTrP with greater intensity of pain, function, quality of life, and sleep quality [26].

In fact, active MTrPs in the sartorius muscle are as prevalent (11.1%) as in other knee muscles such as gastrocnemius, vastus lateralis, and medialis [7,8]. Surprisingly, the presence of active MTrPs in other muscles more commonly associated with anterior knee pain, e.g., gracilis, tibialis anterior, and rectus femoris, have shown a prevalence rate of 5.5%. Itoh et al. [12] reported a prevalence rate of 20% for active MTrPs in this muscle. Nevertheless, several muscles including quadriceps (60%), iliopsoas, and adductors (40%) showed greater prevalence rates [7,8,12].

Despite this high prevalence of active MTrPs located at the sartorius muscle in clinical populations, the number of experimental studies analyzing the treatment effects of targeting this muscle is limited. Itoh et al. [12] demonstrated higher improvements in pain intensity and function in individuals with knee osteoarthritis as compared with sham dry needling and standard acupuncture. However, although treatments based on MTrP management at this location seem to be reasonable and effective in improving clinical outcomes, evidence is limited and some caution should be exercised. For instance, further studies with larger samples are needed to confirm the association between the prevalence of MTrPs in the sartorius muscle and clinical outcomes and their role in the etiology.

One likely reason for this lack of evidence may be related to a reluctance by clinicians to perform invasive treatments nearby the femoral triangle. Although the femoral neurovascular bundle (consisting of nerve, artery, vein, and lymph tissue) can be avoided with relative safety by palpating the femoral artery and then moving laterally to it to insert the needle, the locations assessed in this study present a higher risk of vascular puncture as the artery is normally located deep in the sartorius muscle and selecting a proper needle length is needed [16]. Although the only way to assure the most appropriate length is the use of ultrasound imaging (which is increasing in popularity), its use is not always possible and the developed model in the current study can significantly improve patients’ safety (e.g., preventing bleeding and vasovagal responses) [18].

### Limitations

Although this study has shown promising results, potential limitations should be recognized. First, this prediction model was based on a sample of healthy subjects, and therefore morphological changes in clinical populations may bias the accuracy of the model. Further research should include clinical pain populations to determine if different variables contribute to the model. Second, there are at least two variants reported in the literature affecting the structure of the femoral nerve and the anatomical variability in branches arising from the femoral artery. Finally, this prediction model was developed to assist clinicians during needling procedures based on “easy-to-assess” variables and its accuracy is limited for this reason (other potential variables improving the accuracy of the model were not considered because of the difficulty of measurement), but it should not be considered as a potential replacement for ultrasound-guided needling.

## 5. Conclusions

This study found that sartorius muscle deep limit assessed with ultrasound in healthy subjects can be predicted with 51.1% accuracy at the proximal third and 42.6% at the mid-third based on demographic and anthropometric features. Gender, proximal-third thigh girth, and BMI were the most relevant features for sartorius muscle lower limit depth. Although anatomical variants for the femoral artery are possible, this applicable regression model could assist clinicians in estimating the most appropriate needle length needed during the needling of the sartorius muscle to reduce the risk of adverse effects.

## Figures and Tables

**Table 1:** Participants’ baseline demographic characteristics.

Variables	Sample (*n* = 84)	Males (*n* = 49)	Females (*n* = 35)
Age (years) †	24.1 ± 8.2	25.9 ± 9.6	21.6 ± 4.8
Height (m) *	1.73 ± 0.09	1.78 ± 0.07	1.65 ± 0.06
Weight (kg) *	71.2 ± 14.5	77.6 ± 13.5	62.2 ± 10.6
BMI (kg/m^2^) †	23.6 ± 3.6	24.3 ± 3.6	22.5 ± 3.3

* Significant between-gender differences (*p* < 0.001). † Significant between-gender differences (*p* < 0.05).

**Table 2:** Anthropometric and ultrasonographic features by gender and leg side.

Variables	Gender			Leg Side		
	Males (*n* = 49)	Females (*n* = 35)	Difference	Left (*n* = 84)	Right (*n* = 84)	Difference
Thigh Length (cm)	57.6 ± 3.4	54.3 ± 3.1	*3.2 (1.8; 4.7) p* < *0.001*	56.1 ± 3.6	56.2 ± 3.6	*0.9 (−1.2; 1.0) p* = *0.87*
Thigh Girth (cm)						
*Proximal Third*	60.1 ± 6.7	58.4 ± 7.6	*1.7 (−1.4; 4.9) p* = *0.27*	59.3 ± 6.6	59.4 ± 7.1	*0.1 (−2.1; 2.1) p* = *0.98*
*Mid-Third*	54.9 ± 6.6	52.6 ± 7.9	*2.3 (−0.9; 5.5) p* = *0.15*	53.8 ± 7.1	54.0 ± 7.2	*0.2 (−2.3; 2.0) p* = *0.88*
Sartorius Depth (mm)						
*Proximal Third*	26.9 ± 7.3	31.6 ± 6.2	*4.6 (1.6; 7.6) p* = *0.003*	28.9 ± 6.7	28.9 ± 7.2	*0.0 (−2.1; 2.1) p* = *0.98*
*Mid-Third*	30.5 ± 7.1	34.8 ± 5.6	*4.3 (1.4; 7.2) p* = *0.004*	32.0 ± 6.8	32.3 ± 6.8	*0.3 (−1.7; 2.4) p* = *0.73*

Baseline values are expressed as Mean ± Standard Deviation; Between Groups Differences are expressed as Mean (95% Confidence Interval) and *p*-Values.

**Table 3:** Pearson-Product Moment Correlation Matrix.

	1	2	3	4	5	6	7	8	9	10
1. Thigh Length										
2. Proximal-Third Girth	0.183 *									
3. Mid-Third Girth	0.339 **	0.430 **								
4. Gender	−0.444 **	n.s.	n.s.							
5. Age	n.s.	n.s.	n.s.	−0.257 **						
6. Weight	0.840 **	0.199 **	0.253 **	−0.656 **	n.s.					
7. Height	0.603 **	0.614 **	0.705 **	−0.527 **	−0.208 **	0.660 **				
8. BMI	0.209 **	0.656 **	0.757 **	−0.249 **	0.261 **	0.173 *	0.848 **			
9. Side	n.s.	n.s.	n.s.	n.s.	n.s.	n.s.	n.s.	n.s.		
10. Proximal-Third Sartorius Depth	n.s.	0.520 **	0.382 **	0.361 **	n.s.	n.s.	0.305 **	0.470 **	n.s.	
11. Mid-Third Sartorius Depth	n.s.	0.505 **	0.293 **	0.316 **	n.s.	n.s.	0.284 **	0.422 **	n.s.	0.771 **

n.s. non-significant; * *p* < 0.05; ** *p* < 0.01.

**Table 4:** Summary of the Regression Analyses to determine Predictors of Sartorius Depth.

	Predictor Outcome	B	SE B	95% CI	β	t	p
Proximal-Third	Step 1						
	Gender	0.508	0.102	(0.306; 0.709)	0.361	4.972	<0.001
	Step 2						
	Gender	0.581	0.083	(0.418; 0.744)	0.413	7.038	<0.001
	Proximal-Third Girth	0.057	0.006	(0.045; 0.068)	0.559	9.519	<0.001
	Step 3						
	Gender	0.684	0.079	(0.528; 0.841)	0.487	8.643	<0.001
	Proximal-Third Girth	0.032	0.007	(0.017; 0.046)	0.311	4.310	<0.001
	BMI	0.075	0.014	(0.046; 0.103)	0.387	5.211	<0.001
Mid-Third	Step 1						
	Gender	0.438	0.103	(0.236; 0.641)	0.316	4.275	<0.001
	Step 2						
	Gender	0.508	0.085	(0.340; 0.676)	0.366	5.968	<0.001
	Proximal-Third Girth	0.054	0.006	(0.042; 0.066)	0.540	8.797	<0.001
	Step 3						
	Gender	0.587	0.085	(0.420; 0.755)	0.423	6.929	<0.001
	Proximal-Third Girth	0.035	0.008	(0.019; 050)	0.349	4.453	<0.001
	BMI	0.057	0.015	(0.027; 087)	0.299	3.711	<0.001

Proximal-third sartorius depth: R2 adj. = 0.125 for step 1 (F = 24.725; *p* < 0.001), R2 adj. = 0.433 for step 2 (F = 64.377; *p* < 0.001), R2 adj. = 0.511 for step 3 (F = 58.816; *p* < 0.001). Mid-third sartorius depth: R^2^ adj. = 0.100 for step 1 (F = 18.272; *p* < 0.001), R^2^ adj. = 0.388 for step 2 (F = 52.061; *p* < 0.001), R^2^ adj. = 0.426 for step 3 (F = 42.001; *p* < 0.001).

## Data Availability

All data derived from this study are presented in the article.

## Supplementary Materials

The following are available online at https://www.mdpi.com/article/10.3390/tomography8050208/s1, Table S1: Alternative Regression Analyses to determine Predictors of Sartorius Depth.
