# Supplementary material for: Regression Model Decreasing the Risk of Femoral Neurovascular Bundle Accidental Puncture
Source: Tomography. 2022 Oct 1;8(5):2498–507. doi: 10.3390/tomography8050208 (PMC9611046; doi:10.3390/tomography8050208)
Supplement: Supplementary file 1 [file tomography-08-00208-s001.zip › tomography-1929540-supplementary.pdf]

Supplementary Material. Alternative Regression Analyses to determine Predictors of Sartorius Depth.

	Predictor Outcome	*B*	SE B	95% CI	*β*	*t*	*P*
Proximal-Third	Step 1						
	Gender	0.508	0.102	(0.306; 0.709)	0.361	4.972	<0.001
	Step 2						
	Gender	0.581	0.083	(0.418; 0.744)	0.413	7.038	<0.001
	Proximal-Third Girth	0.057	0.006	(0.045; .0068)	0.559	9.519	<0.001
	Step 3						
	Gender	0.648	0.110	(0.431; 0.864)	0.461	5.908	<0.001
	Proximal-Third Girth	0.056	0.006	(0.044; 0.068)	0.549	9.181	<0.001
	Height	0.545	0.587	(-0.614; 1.705)	0.074	0.929	0.354
Mid-Third	Step 1						
	Gender	0.438	0.103	(0.236; 0.641)	0.316	4.275	<0.001
	Step 2						
	Gender	0.508	0.085	(0.340; 0.676)	0.366	5.968	<0.001
	Proximal-Third Girth	0.054	0.006	(0.042; 0.066)	0.540	8.797	<0.001
	Step 3						
	Gender	0.558	0.113	(0.334; 0.782)	0.402	4.926	<0.001
	Proximal-Third Girth	0.053	0.006	(0.041; 0.066)	0.532	8.509	<0.001
	Height	0.406	0.607	(-0.792; 1.604)	0.056	0.669	0.504

Proximal-third sartorius depth: R^2 adj. = 0.125 for step 1 (F = 24.725; p<0.001), R^2 adj. = 0.433 for step 2 (F = 64.377; p<0.001), R^2 adj. = 0.432 for step 3 (F = 43.170; p<0.001).
Mid-third sartorius depth: R^2 adj. = .100 for step 1 (F = 18.272; p<0.001), R^2 adj. = 0.388 for step 2 (F = 52.061; p<0.001), R^2 adj. = 0.390 for step 3 (F = 34.740; p<0.001).
